# Haploinsufficiency of vascular endothelial growth factor related signaling genes is associated with tetralogy of Fallot

**DOI:** 10.1038/s41436-018-0260-9

**Published:** 2018-09-20

**Authors:** Miriam S. Reuter, Rebekah Jobling, Rajiv R. Chaturvedi, Roozbeh Manshaei, Gregory Costain, Tracy Heung, Meredith Curtis, S. Mohsen Hosseini, Eriskay Liston, Chelsea Lowther, Erwin Oechslin, Heinrich Sticht, Bhooma Thiruvahindrapuram, Spencer van Mil, Rachel M. Wald, Susan Walker, Christian R. Marshall, Candice K. Silversides, Stephen W. Scherer, Raymond H. Kim, Anne S. Bassett

**Affiliations:** 1 Ted Rogers Centre for Heart Research, Cardiac Genome Clinic, The Hospital for Sick Children, Toronto, Ontario Canada; 2 The Centre for Applied Genomics, The Hospital for Sick Children, Toronto, Ontario Canada; 3 Division of Clinical and Metabolic Genetics, The Hospital for Sick Children, Toronto, Ontario Canada; 4 Genome Diagnostics, Department of Paediatric Laboratory Medicine, The Hospital for Sick Children, Toronto, Ontario Canada; 5 Labatt Heart Centre, Division of Cardiology, The Hospital for Sick Children, Toronto, Ontario Canada; 6 Clinical Genetics Research Program, Centre for Addiction and Mental Health, Toronto, Ontario Canada; 7 Division of Cardiology, Toronto Congenital Cardiac Centre for Adults at the Peter Munk Cardiac Centre, Department of Medicine, University Health Network, Toronto, Ontario Canada; 8 Institute of Biochemistry, Friedrich-Alexander-Universität Erlangen-Nürnberg, Erlangen, Germany; 9 Program in Genetics and Genome Biology, The Hospital for Sick Children, Toronto, Ontario Canada; 10 Centre for Genetic Medicine, The Hospital for Sick Children, Toronto, Ontario Canada; 11 Laboratory Medicine and Pathobiology, University of Toronto, Toronto, Ontario Canada; 12 Department of Molecular Genetics, University of Toronto, Toronto, Ontario Canada; 13 Fred A. Litwin Family Centre in Genetic Medicine, University Health Network, Department of Medicine, University of Toronto, Toronto, Ontario Canada; 14 The Dalglish Family 22q Clinic for Adults with 22q11.2 Deletion Syndrome, Department of Psychiatry, and Toronto General Research Institute, University Health Network, Toronto, Ontario Canada; 15 Department of Psychiatry, University of Toronto, Toronto, Ontario Canada

**Keywords:** tetralogy of Fallot, genome sequencing, VEGF, *FLT4*, haploinsufficiency, congenital heart disease, conotruncal defects

## Abstract

**Purpose:**

To determine disease-associated single-gene variants in conotruncal defects, particularly tetralogy of Fallot (TOF).

**Methods:**

We analyzed for rare loss-of-function and deleterious variants in *FLT4* (VEGFR3) and other genes in the vascular endothelial growth factor (VEGF) pathway, as part of a genome sequencing study involving 175 adults with TOF from a single site.

**Results:**

We identified nine (5.1%) probands with novel *FLT4* variants: seven loss-of-function, including an 8-kb deletion, and two predicted damaging. In ten other probands we found likely disruptive variants in VEGF-related genes: *KDR* (VEGFR2; two stopgain and two nonsynonymous variants), *VEGFA*, *FGD5*, *BCAR1*, *IQGAP1*, *FOXO1*, and *PRDM1*. Detection of VEGF-related variants (19/175, 10.9%) was associated with an increased prevalence of absent pulmonary valve (26.3% vs. 3.4%, *p* < 0.0001) and right aortic arch (52.6% vs. 29.1%, *p* = 0.029). Extracardiac anomalies were rare. In an attempt to replicate findings, we identified three loss-of-function or damaging variants in *FLT4*, *KDR*, and *IQGAP1* in ten independent families with TOF.

**Conclusion:**

Loss-of-function variants in *FLT4* and *KDR* contribute substantially to the genetic basis of TOF. The findings support dysregulated VEGF signaling as a novel mechanism contributing to the pathogenesis of TOF.

## Introduction

Tetralogy of Fallot (TOF) is the most common cyanotic heart malformation in humans. Approximately 20% of TOF patients are diagnosed with genetic syndromes.^1^ Recurrent 22q11.2 deletions, associated with 22q11.2 deletion syndrome, and other rare copy-number variants (CNVs) contribute substantially to the genetic burden, and have suggested disease-related mechanisms, such as disturbances of cell migration and vasculature development.^2^ The role of genetic factors is further supported by an increased risk of congenital heart defects (CHD) in first-degree relatives of TOF patients.^3^ However, for the majority of individuals with TOF, the etiology remains unknown. TOF-associated single-gene defects are rarely identified. A multisite collaborative study using exome sequencing recently identified *FLT4* loss-of-function variants in 2.3% of children with TOF.^4^ Exome sequencing also revealed another *FLT4* frameshift deletion in a TOF patient.^5^ As part of a genome sequencing study of the underlying genetic causes in adults with CHD, predominantly TOF, from a single site, we investigated rare and predicted damaging variants in *FLT4* and other vascular endothelial growth factor (VEGF)-related genes.

## Materials and methods

### Study participants

The study was approved by the Research Ethics Boards at the University Health Network (REB 98-E156), Centre for Addiction and Mental Health (REB 154/2002), and The Hospital for Sick Children (REB 1000053844). Informed consent was obtained from all probands and/or their legal guardians.

*Cohort 1:* Study participants with microarray data available were selected from a well-characterized cohort of *n* = 552 unrelated adults with TOF or related congenital heart defects and no 22q11.2 microdeletion, recruited from the Toronto Congenital Cardiac Centre for Adults.^2,6^ We performed genome sequencing on *n* = 231 probands (175 TOF, 49 transposition of the great arteries, 7 other CHD). Of these, by design, *n* = 122 (92 TOF) had no rare (<0.1%) genic CNVs >10 kb, whereas *n* = 109 (83 TOF) had rare, autosomal CNVs >10 kb overlapping putative CHD candidate genes (Supplementary information, Tables S3 and S6, contain details on design and selection for sequencing).^2,6^

*Cohort 2:* We additionally performed genome sequencing of 11 individuals with TOF from ten families, eight of which were sequenced as parent–child trios. The families originated from a larger cohort of various CHD, recruited through the Ted Rogers Cardiac Genome Clinic.

### Genome sequencing

DNA was sequenced on the Illumina HiSeq X system at The Centre for Applied Genomics (TCAG) in Toronto, Canada (Supplementary information, Table S1).^7^ Population allele frequencies were derived from 1000 Genomes, ExAC, and gnomAD (Supplementary information). Probability of loss-of-function intolerance (pLI) scores were derived from ExAC (http://exac.broadinstitute.org/); haploinsufficiency (HI) predictions were derived from DECIPHER (https://decipher.sanger.ac.uk/).

## Results

### Rare *FLT4* variants associated with tetralogy of Fallot

**Fig. 1:**
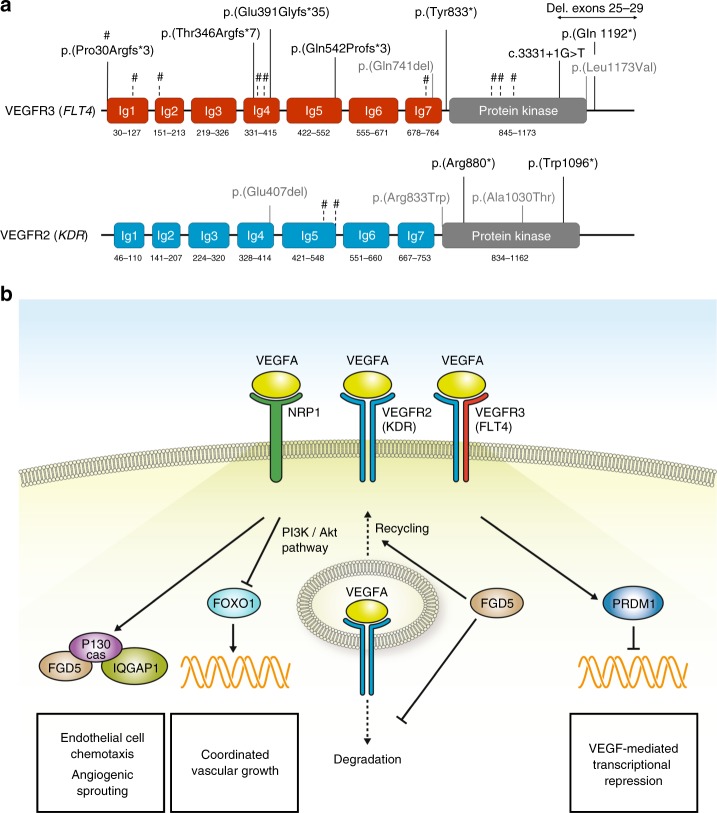
VEGF pathway and genome sequencing in tetralogy of Fallot. (**a**) Variant positions in vascular endothelial growth factor receptors 3 (VEGFR3; *FLT4*) and 2 (VEGFR2; *KDR*): loss-of-function variants (black; multiexon 8-kb deletion indicated by horizontal arrow), in-frame deletions or deleterious missense variants (gray). Loss-of-function variants in ref.^4^ indicated by vertical dashed lines and #; in *FLT4* (NM_182925.4), from left to right: p.(Pro30Argfs*3) [1x inherited, 1x de novo], p.(Arg82*), p.(Thr168Serfs*76), p.(Tyr361*), p.(Pro364Alafs*63), p.(Gln736*), p.(Leu935Profs*72), p.(Cys949Argfs*53), p.(Gln999*); and in *KDR* (NM_002253.2): p.(Lys529*), c.1646-2A>T. Nomenclature as recommended by the Human Genome Variation Society (HGVS; http://varnomen.hgvs.org/). (**b**) Selected components of vascular endothelial growth factor (VEGF) signaling in endothelial cells, focusing on candidate genes for tetralogy of Fallot and their presumed roles in vascular development. VEGFA induces the formation of VEGFR2 homodimers (blue/blue), VEGFR2/ VEGFR3 heterodimers (blue/red), and binds to the coreceptor NRP1 (ref. ^9^). VEGFR1 (encoded by *FLT1*; not shown) may function as a negative regulator for VEGFA signaling, but also forms heterodimers with VEGFR2 (ref. ^9^). P130cas (encoded by *BCAR1*) mediates VEGFR2/NRP1 signaling and functions in the assembly of multiprotein complexes, among which are IQGAP1 and FGD5 (ref. ^17^). FGD5 also inhibits VEGFR2 degradation.^18^ VEGFR2 suppresses the activity of the transcription factor FOXO1, which is important for the regulation of coordinated vascular sprouting.^19^ The transcriptional repressor PRDM1 was linked to VEGF signaling in tumor vasculature and in wound healing;^20^ arterial pole defects in mutant mice indicate PRDM1 also functions in cardiovascular development (Table S5)

As an initial stage of this study on adults with congenital cardiac disease, we investigated genome sequencing data for disease-associated single-nucleotide variants (SNVs) and CNVs in the VEGF pathway. We identified nine previously unreported variants in *FLT4*, encoding vascular endothelial growth factor receptor 3 (VEGFR3). All were within the 175 individuals with TOF, thus the prevalence of *FLT4* variants in this adult TOF cohort was 5.1% (9/175). Seven of the variants had loss-of-function effects (two stopgain, three frameshift insertion/deletions, one canonical splice site, one multiexon 8-kb deletion; Fig. 1a and Table 1). A missense variant p.(Leu1173Val) was predicted to be deleterious (CADD = 25, SIFT = 0, PolyPhen2 = 1), and was located in the terminal α-helix of the protein kinase domain, adjacent to a cluster of phosphorylated residues. An in-frame deletion p.(Glu741del) in immunoglobulin homology domain 7 (Ig7), close to the dimerization site Arg737 (ref. ^8^), was predicted to impact affinity for dimer formation. None of the nine individuals were considered syndromic (Table 1). One proband (TOF158) had a daughter with TOF, who had inherited the paternal *FLT4* stopgain variant.

**Table 1:** Individuals with tetralogy of Fallot and likely disruptive variants in genes in the vascular endothelial growth factor pathway

Case^a^	Sex	Age^b^ (years)	Phenotype and family history of CHD	Gene (transcript)	Variant type	Variant	Chromosomal position (GRCh37/hg19)	Allele frequency (ExAC/gnomAD)^c^	Other variants of uncertain significance^d^	
									SNVs	CNVs
**Cohort 1 (*n*** **=** **19 individuals)**										
TOF293	F	23	TOF, RAA, APV; stillborn offspring	*FLT4* (NM_182925.4)	Deletion (multiexon)	Deletion of exons 25–29	chr5:g.[180031767_180040470del]	0 / 0	●	
TOF158	M	79	TOF, RAA, paroxysmal atrial flutter requiring ablation, mild aortic dilatation; depression and/or anxiety, migraine, melanoma; daughter with TOF^**e**^	*FLT4* (NM_182925.4)	Stopgain	c.3574C>T, p.(Gln1192*)	chr5:180038443G>A	0 / 0	●	
TOF238	M	42	TOF, RAA, MAPCA, PA; aortic dilatation	*FLT4* (NM_182925.4)	Stopgain	c.2499C>G, p.(Tyr833*)	chr5:180047216G>C	0 / 0	●	●
TOF284	M	29	TOF, MAPCA, inconclusive results about RAA; aortic valve replacement	*FLT4* (NM_182925.4)	Duplication (frameshift)	c.1622dupG, p.(Gln542Profs*3)	chr5:180049766dupC	0 / 0		
TOF254	F	32	TOF, APV; bilateral femoral vein occlusions; depression and/or anxiety	*FLT4* (NM_182925.4)	Deletion (frameshift)	c.1172_1173delAG, p.(Glu391 Glyfs*35)	chr5:180053196delCT	0 / 0		●
TOF68	F	20	TOF, RAA, APV; depression and/or anxiety	*FLT4* (NM_182925.4)	Deletion (frameshift)	c.1037delC, p.(Thr346Argfs*7)	chr5:180055948delG	0 / 0		●
TOF301	F	29	TOF, RAA, paternal first cousin with suspected VSD	*FLT4* (NM_182925.4)	Splice site	c.3331+1G>T, p.?	chr5:180041067C>A	0 / 0	●	
TOF271	M	39	TOF, obesity	*FLT4* (NM_182925.4)	Missense	c.3517C>G, p.(Leu1173Val)	chr5:180039526G>C	0 / 0		
TOF236	F	33	TOF, RAA; atrioventricular nodal reentry tachycardia requiring ablation; depression and/or anxiety; unilateral duplicated ureter; daughter with truncus arteriosus	*FLT4* (NM_182925.4)	Deletion (in-frame)	c.2223_2225delGGA, p.(Glu741del)	chr5:180047950delTCC	0 / 0		
TOF109	M	44	TOF, PFO or ASD, atrial flutter; obesity; mild cognitive and memory problems attributed to cerebral ischemia; brother died in infancy of suspected cyanotic CHD	*KDR* (NM_002253.2)	Stopgain	c.3287G>A, p.(Trp1096*)	chr4:55955875C>T	0 / 0		
TOF155	M	52	TOF, PFO or ASD; depression and/or anxiety; gastroesophageal reflux	*KDR* (NM_002253.2)	Stopgain	c.2638C>T, p.(Arg880*)	chr4:55962486G>A	0 / 0	●	●
TOF326	F	46	TOF, RAA, PFO or ASD; short stature; benign brain tumor	*KDR* (NM_002253.2)	Missense	c.2497C>T, p.(Arg833Trp)	chr4:55964316G>A	0 / 0		
TOF359	F	30	TOF, APV; learning difficulties; maternal uncle with unspecified cyanotic CHD	*KDR* (NM_002253.2)	Deletion (in-frame)	c.1219_1221delGAG, p.(Glu407del)	chr4:55976604delCTC	0 / 0		
TOF241	M	29	TOF, RAA, bicuspid pulmonic valve; short stature, obesity; learning difficulties; depression and/or anxiety; stillborn offspring	*VEGFA* (NM_001171623.1)	Stopgain	c.115G>T, p.(Glu39*)	chr6:43742126G>T	0 / 0		
TOF89	M	53 (died 55)	TOF, PFO or ASD; inducible atrial flutter/fibrillation, systemic arterial hypertension, aortic dilatation, query BAV; ankylosing spondylitis	*FGD5* (NM_152536.3)	Stopgain	c.3673C>T, p.(Arg1225*)	chr3:14963921C>T	0 / 0	●	
TOF220	F	32	TOF, RAA, APV; learning difficulties	*BCAR1* (NM_001170715.1)	Deletion (multiexon)	Deletion of exons 2–7	chr16:g.[75237177_75301117del]	0 / 0	●	●
TOF48	F	26	TOF, PFO or ASD, bicuspid pulmonic valve	*IQGAP1* (NM_003870.3)	Stopgain	c.309C>G, p.(Tyr103*)	chr15:90969495C>G	0 / 0		●
TOF62	M	54	TOF, RAA; learning difficulties	*FOXO1* (NM_002015.3)	Deletion (frameshift)	c.580_586delGTGCCCT, p.(Val194Thrfs*137)	chr13:41239764delAGGGCAC	0 / 0		●
TOF53	F	52	TOF, PFO or ASD; coronary artery bypass grafts, systemic arterial hypertension; depression and/or anxiety	*PRDM1* (NM_001198.3)	Stopgain	c.1824C>A, p.(Cys608*)	chr6:106554296C>A	0 / 0		●
**Cohort 2 (*n*** **=** **3 families)**										
CGC-034	F	1	TOF, PA, MAPCA; lymphedema; maternal grandfather with bradycardia	*FLT4* (NM_182925.4)	Deletion (frameshift)	c.89delC, p.(Pro30Argfs*3)	chr5:180058748delG	0 / 4.12e-6		
CGC-001	F, F	1, 24	TOF, PA, absent central pulmonary arteries, MAPCA; accessory bronchus; wide nasal bridge, broad nasal tip, downturned corners of the mouth, clinodactyly, short thumb; mother with TOF, PA, mild intellectual disability	*KDR* (NM_002253.2)	Missense	c.3088G>A, p.(Ala1030Thr)	chr4:55956227C>T	0 / 0	●	
CGC-076	M	1	TOF with severe PS, confluent pulmonary arteries, DORV, bilateral SVC (left SVC to the coronary sinus); esophageal atresia with tracheal fistula, bilateral inferior iris coloboma, clinodactyly of all fifth digits; short stature	*IQGAP1* (NM_003870.3)	Stopgain	c.2296 C > T, p.(Arg766*)	chr15:91016189 C > T	0 / 4.06e-6	●	

All variants are heterozygous. Subjects in this table are of European descent, by design for cohort 1. No subject in cohort 1 had lymphedema or intellectual disability documented. Obesity was defined as body mass index (BMI) consistently >30 as an adult. Short stature was defined as height <3rd percentile using standard adult growth curves. Prevalence of liveborn offspring with major CHD in the adult cohort of 19 patients with TOF: 2/17 (11.8%), plus two stillborn offspring; prevalence of siblings with major CHD 1/40 (2.5%). The median age at TOF repair was 4 years (range 1–22) for this adult cohort of median age 33 (range 26–79) years.

*APV,* absent pulmonary valve; *ASD*, atrial septal defect; *BAV*, bicuspid aortic valve; *CHD*, congenital heart disease; *CNV*, copy-number variant; *DORV*, double outlet right ventricle; *F*, female; *M*, male; *MAPCA*, major aortopulmonary collateral arteries; *PA*, pulmonary atresia; *PFO*, patent foramen ovale; *PS*, pulmonary stenosis; *RAA*, right aortic arch; *SNV*, single-nucleotide variant; *SVC*, superior vena cava/cavae; *TOF*, tetralogy of Fallot; *VSD*, ventricular septal defect.

^a^Case numbers for cohort 1 are those used for the same subjects in a previous report.^2^

^b^Age at last follow-up.

^c^As of March 2018 for both ExAC and gnomAD databases (by design, allele frequencies in ExAC were null for cohort 1).

^d^See Methods re study design with respect to CNVs, and Table S3 for details of putative CHD-related CNVs and SNVs identified.

^e^Inherited paternal *FLT4* variant (TOF158)

### Variants in other vascular endothelial growth factor related genes

Assessing for rare variants in other genes encoding vascular endothelial growth factors (*VEGFA*, *VEGFB*, *VEGFC*, *VEGFD*, *PGF*) or their receptors (*FLT1*, *KDR*, *NRP1*, *NRP2*),^9^ we identified two stopgain and two nonsynonymous variants in *KDR* (encoding VEGR2; Fig. 1a and Table 1), and a stopgain variant p.(Glu39*) in *VEGFA* predicted to affect all isoforms. All variants were identified in individuals with TOF and absent in public databases. Like *FLT4*, both *KDR* and *VEGFA* were predicted to be intolerant to loss-of-function variants (*KDR*: pLI = 0.98, HI = 2.2%; *VEGFA*: pLI = NA, HI = 0.1%). The *KDR* missense variant p.(Arg833Trp) was predicted to be deleterious (CADD = 33, SIFT = 0, PolyPhen2 = 1), potentially through a disruption of the terminal protein kinase structure. The in-frame deletion p.(Glu407del) was in Ig4, a domain important for receptor activity and signaling.^10^

Under the hypothesis that haploinsufficiency of the VEGF signaling pathway is associated with TOF, and causative genes are likely intolerant to loss-of-function variation, we then systematically analyzed the data set for such variants. We screened unreported stopgain, frameshift, and canonical splice-site variants (*n* = 105) and coding deletions (*n* = 13), in 3230 genes with ExAC pLI >0.9 for known functions in the VEGF signaling pathway (Supplementary information). Thereby we identified five additional null variants (Fig. 1b and Table 1): a stopgain variant p.(Arg1225*) in *FGD5* (pLI = 0.99), a deletion of exons 2–7 in *BCAR1* (pLI = 0.99), a stopgain variant p.(Tyr103*) in *IQGAP1* (pLI = 1), a frameshift deletion p.(Val194Thrfs*137) in *FOXO1* (pLI = 0.97), and a stopgain variant p.(Cys608*) in *PRDM1* (pLI = 0.98).

### Clinical phenotype

Nineteen (nine males, 10 females) of 175 (10.9%) probands with TOF were identified with VEGF pathway-associated variants. Individuals with VEGF-related variants and TOF were enriched for absent pulmonary valve: 5/19 (26.3%) vs. 6/175 (3.4%) (Fisher’s exact test; FET: *p* < 0.0001, odds ratio 52.4, 95% confidence interval [5.4–2586.4]) and right aortic arch: 10/19 (52.6%) vs. 51/175 (29.1%) (FET: *p* = 0.029, odds ratio 3.1, 95% confidence interval [1.05–9.3]). None had lymphedema. We did not identify any other likely causal variants in these 19 probands. However, eight (42.1%) of the 19, including three with *FLT4* variants, were amongst those with putative CHD-relevant CNVs. Phenotypic information and additional rare variants are summarized in Tables 1 and S3.

### Additional cohorts

Using genome sequencing data for another cohort (*n* = 11 individuals with TOF from ten families), we discovered three other variants in VEGF pathway genes. In a patient with TOF and congenital lymphedema, we identified a previously described frameshift variant p.(Pro30Argfs*3) in *FLT4* (ref. ^4^), inherited from her mother with normal echocardiography results. We identified a predicted damaging *KDR* missense variant p.(Ala1030Thr) (CADD = 35, SIFT = 0, PolyPhen2 = 1), located in the protein kinase domain adjacent to the catalytic residues Asp1028 and Arg1032 (ref. ^11^) in a mother and daughter, both with TOF and pulmonary atresia. In a patient with complex congenital cardiac disease including TOF (Table 1), esophageal atresia with tracheal fistula, bilateral iris coloboma, and clinodactyly of all fifth digits, we identified another stopgain variant p.(Arg766*) in *IQGAP1*, inherited from his unaffected father.

Review of previously published microarray studies revealed several *FLT4* and other VEGF-related genes impacted by rare CNVs in individuals with cardiac defects (Table S4). Apart from one frameshift insertion in *BCAR1*, there were no rare loss-of-function variants of *FLT4*, *KDR*, *VEGFA*, *FGD5*, *IQGAP1*, *FOXO1*, or *PRDM1* identified in the genome sequencing data of 7231 individuals with autism from the MSSNG database (https://www.mss.ng/#).

## Discussion

Our results support the hypothesis that dysregulated VEGF signaling contributes to the genetic etiology of TOF. We confirmed the importance of deleterious *FLT4* variants,^4^ and identified null alleles in multiple haploinsufficiency-intolerant genes in the VEGF pathway.

For *FLT4* variants, the results were overall consistent with a previous study^4^ that reported similar variants in 9 of 426 nonsyndromic TOF probands and one subject with an unspecified conotruncal defect, but no association with neurodevelopmental disorders or other congenital anomalies. *FLT4* variants were more prevalent in our cohort than in the previous report (5.1% vs. 2.3%) (ref. ^4^). There could be several reasons for this beyond sampling variability. Genome sequencing results in more uniform and complete coverage of coding regions than exome sequencing, and enables the detection of structural variants (e.g., small CNVs, such as those identified in *FLT4* and *BCAR1*; Fig. S1). Another difference in study design was that the adult cohort studied here had undergone extensive microarray studies, although we found no evidence to support enrichment for disease-associated single-gene defects in the *n* = 92 (52.6%) TOF patients with no cardiac disease–related rare CNVs (Table S3). Our analysis also considered missense variants and in-frame deletions/insertions, in addition to obvious loss-of-function alleles examined in the previous exome sequencing study.^4^

None of the loss-of-function *FLT4* variants identified through genome sequencing in our adult TOF cohort overlapped with those previously reported.^4^ However, we identified one previously reported,^4^ recurrent frameshift deletion (Fig. 1a) in an infant with both TOF and lymphedema. Missense variants in the protein kinase domain reported to cause Milroy disease (hereditary lymphedema, OMIM-P 153100) provide evidence for allelic heterogeneity in *FLT4*. However, robust genotype–phenotype correlations are challenged by the abovementioned frameshift deletion and by a missense substitution in the protein kinase domain in an individual with isolated TOF (Fig. 1a). The absence of lymphedema history in our adult cohort, including those with *FLT4* variants, would suggest at most a mild or fully remitted lymphedema phenotype.

The case-only adult cohort design did not allow for systematic segregation testing in family members; this will be the focus of future studies. However, as for most (6/9) families with incomplete penetrance of *FLT4*-associated TOF in a previous study,^4^ an *FLT4* variant in our second TOF cohort was inherited from an unaffected mother. This evidence for reduced penetrance and variable expression may be related to other, as yet unidentified, genetic and perhaps nongenetic factors relevant to expression of the TOF phenotype, such as oligogenic inheritance models. Estimating recurrence risks and penetrance will require larger disease and population-based cohorts.

*FLT4* encodes VEGFR3, one of three main cell surface receptors for vascular endothelial growth factors. We conjectured that variants in other genes involved in VEGF signaling could disrupt this network and may also be involved in the etiopathogenesis of TOF. We identified *KDR* (encoding VEGFR2) as a novel TOF-associated gene, with five novel damaging variants in our data set (Fig. 1a). This was supported by loss-of-function variants in two individuals with conotruncal defects reported in supplementary data of a previous study.^4^ We also detected a stopgain variant in *VEGFA* as a further candidate for TOF pathogenesis. *VEGFA* perturbation has previously been linked to cardiac development and TOF.^12,13^ VEGFA and VEGFR2 are the best studied regulators of vascular development under physiological and pathological conditions. VEGFA induces the formation of VEGFR2 homodimers and VEGFR2/VEGFR3 heterodimers, both of which are involved in the regulation of angiogenic sprouting.^9,14^

Our analyses identified null alleles in additional candidate genes that link the VEGF signaling pathway to TOF: *FGD5*, *BCAR1*, *IQGAP1* (2x), *FOXO1*, and *PRDM1* (Fig. 1b and Supplementary information). Mouse constitutive knockout models support a role for these VEGF-related genes in cardiovascular development (Table S5). Mutant *Prdm1* mice show arterial pole defects and pharyngeal arch anomalies that are more severe on a *Tbx1* heterozygous background, reflecting interaction between these two genes. Complete deletion of any of *Flt4*, *Kdr*, *Vegfa*, *Fgd5*, *Bcar1*, or *Foxo1* is embryonically lethal with impaired cardiac and/or vessel development.

We found that TOF probands with VEGF-related variants were enriched for the presence of absent pulmonary valve and right aortic arch. Impairment of asymmetric VEGF signaling and blood flow were previously linked to right aortic arch.^15^ Further studies are required to confirm that haploinsufficiency of *VEGFA*, *FGD5*, *BCAR1*, *IQGAP1*, *FOXO1*, and *PRDM1* are associated with TOF, and to delineate the associated phenotypes. The functional impacts of the missense and in-frame variants in *FLT4* and *KDR* require elucidation. We did not identify deleterious variants in other promising candidate genes such as *NRP1* (encoding Neuropilin-1, a VEGFR2 coreceptor) or *FLT1* (encoding VEGFR1) in this data set, and statistical evaluation of the VEGF pathway awaits final analyses of all rare variants and gene pathways for the entire cohort sequenced. However, previous studies reported loss-of-function variants in *FLT1* (*n* = 2) or *BCAR1* (*n* = 1) in subjects with conotruncal defects (supplemental data^4^), and a heterozygous deletion encompassing *NRP1* cosegregating with TOF in a single family.^16^

Our findings, in the context of previously published data, support the hypothesis of deficient VEGF signaling as a novel and plausible pathomechanism of TOF and related cardiovascular defects. Loss-of-function variants in *FLT4* and *KDR* contribute substantially to the disease prevalence and warrant consideration for clinical diagnostic testing, particularly in patients with TOF and normal extracardiac development.

## Electronic supplementary material

Supplementary Information

Supplementary Data
