# Supplementary material for: Haploinsufficiency of vascular endothelial growth factor related signaling genes is associated with tetralogy of Fallot
Source: Genet Med. 2018 Sep 20;21(4):1001–7. doi: 10.1038/s41436-018-0260-9 (PMC6752294; doi:10.1038/s41436-018-0260-9)
Supplement: Supplementary file 1 — Supplementary Information [file 41436_2018_260_MOESM1_ESM.docx]

**Supplementary Material for:**

**Haploinsufficiency of vascular endothelial growth factor related signaling genes is associated with tetralogy of Fallot**

Miriam S Reuter, Rebekah Jobling, Rajiv R Chaturvedi, Roozbeh Manshaei, Gregory Costain, Tracy Heung, Meredith Curtis, S Mohsen Hosseini, Eriskay Liston, Chelsea Lowther, Erwin Oechslin, Heinrich Sticht, Bhooma Thiruvahindrapuram, Spencer van Mil, Rachel M Wald, Susan Walker, Christian R Marshall, Candice K Silversides, Stephen W Scherer, Raymond H Kim, Anne S Bassett

**This file includes the following:**

Supplementary information: Study participants, Genome sequencing, Variant calling and annotation, Variant analysis, Statistics.

Table S1. Genome sequencing coverage statistics.

Table S2. Summary descriptive statistics for 19 study participants with tetralogy of Fallot (TOF) and likely disruptive variants in vascular endothelial growth factor (VEGF) related signaling genes (cohort 1).

Table S3. Putative cardiac related variants of uncertain significance in 22 probands with TOF and likely disruptive variants in VEGF related signaling genes.

Figure S1. Multi-exon deletions of *FLT4* and *BCAR1*.

Table S4. Copy number variants overlapping 15 VEGF pathway genes (*BCAR1*, *FGD5*, *FLT1*, *FLT4*, *FOXO1*, *IQGAP1*, *KDR*, *NRP1*, *NRP2*, *PGF*, *PRDM1*, *VEGFA*, *VEGFB*, *VEGFC, or VEGFD)* in individuals with cardiac defects from previous studies.

Supplementary information on the development of the second heart field and vascular endothelial growth factor related genes.

Table S5. Mouse deficiency models of 15 VEGF pathway genes.

Table S6 (Copy number variants in individuals from cohort 1) is provided as a separate document.

**Supplementary information**

**Study participants**

By design, all 231 adult probands with congenital heart disease (CHD) from cohort 1 were unrelated, of European ancestry, and had undergone extensive microarray studies (1, 2). Also by design, a subset of n=109 (47.2%) had rare (<0.1%), autosomal copy number variants (CNVs) >10 kb, that overlapped putative CHD candidate genes (Table S6). These candidate CNVs were identified in previous studies (1, 2), and/or determined from gene sets related to CHD and/or literature supporting a role in heart development (1, 2). Rarity was adjudicated as defined in (1, 2). Of the n=231 probands with genome sequencing data, there were no significant differences between those with (n=109) or without (n=122) rare CNVs in terms of sex (p=0.28), age (p=0.86), or learning difficulties (p=0.88), absent pulmonary valve (p=0.42) or right aortic arch (p=0.20). The selection from n=552 unrelated subjects with CHD was based on ethnicity, the availability of sufficient amount of high quality DNA for sequencing, and to ensure a relative balance in the numbers of those with and without rare CNVs (1, 2).


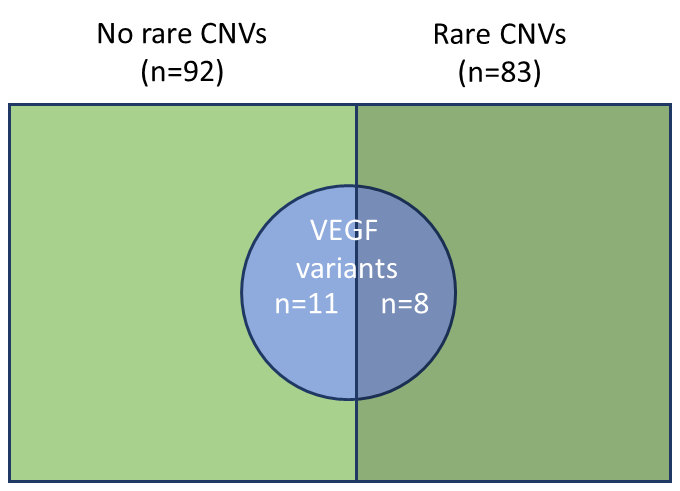


Of n=83 probands with TOF and rare putatively CHD-relevant CNVs, eight (9.6%) were identified with likely disruptive VEGF variants, whereas of n=92 probands with TOF and no rare CNVs, 11 (12.0%) were identified with likely disruptive VEGF variants.

Ten TOF families from cohort 2 were recruited through the Ted Rogers Cardiac Genome Clinic. Of those, six probands had undergone clinical microarray studies (CGC-006, CGC-022, CGC-034, CGC-062, CGC-073, and CGC-076); all 11 probands had CNVs analyzed from genome sequencing data.

**Genome sequencing**

DNA from whole blood (n=238) or saliva was sequenced on the Illumina HiSeq X system at The Centre for Applied Genomics (TCAG) in Toronto, Canada. DNA was quantified using the Qubit High Sensitivity Assay, and sample purity was checked using the Nanodrop OD260/280 ratio. Following the manufacturer’s recommended protocol, 100 ng of DNA were used as input material for library preparation using the Illumina TruSeq Nano DNA Library Prep Kit. In brief, DNA was fragmented to an average of 350 bp by sonication on a Covaris LE220 instrument. Fragmented DNA was end-repaired and A-tailed and indexed TruSeq Illumina adapters added by ligation prior to library amplification. For 231 samples (cohort 1), libraries were amplified by PCR prior to sequencing. For 27 samples (cohort 2, including parents), we used 700 ng of DNA, and the PCR amplification step was omitted to generate “PCR-free” libraries. Libraries were assessed using Bioanalyzer DNA High Sensitivity chips and quantified by quantitative PCR using Kapa Library Quantification Illumina/ABI Prism Kit protocol (KAPA Biosystems). Validated libraries were pooled in equimolar quantities and paired-end sequenced on an Illumina HiSeq X platform following Illumina’s recommended protocol to generate paired-end reads of 150 bases in length.

**Variant calling and annotation**

Base calling was performed using BCL2FASTQ, and data were generated using Illumina HiSeq Analysis Software (HAS; version 2-2.5.55.1311). Reads were mapped to the hg19 reference sequence using the BWA-backtrack algorithm from BWA v0.7.12 (3), and SNV and small indel variants were called using GATK (v3.5-0) according to GATK Best Practices recommendations (4, 5). Variant calls were annotated using a custom pipeline (6, 7) developed at TCAG based on ANNOVAR (8).

CNVs were called using a modified read depth method with the programs ERDS v1.1 (Estimation by Read Depth with Single-nucleotide variants) (9) and CNVnator v0.3.3 (10) using a window size of 500 bp (11, 12). CNV size cut-offs were 1 kb for losses and 2 kb for gains. High-quality CNVs were defined as those detected by ERDS that were also detected by CNVnator with greater than 50% overlap.

**Variant analysis**

1. Predicted loss-of-function alleles

We classified the following as loss-of-function (LoF) or null alleles: frameshift insertions, deletions or substitutions; substitutions creating a stop codon; and alterations of the intronic dinucleotide adjacent to a coding-exonic splice junction.

1. Allele frequency and control databases

Overall and population-specific allele frequencies were derived from 1000 Genomes (African, American, East Asian, European, South Asian; http://www.internationalgenome.org/), NHLBI-ESP (African American, European; http://evs.gs.washington.edu/EVS/), ExAC (African, American, East Asian, Finnish, Non-Finnish Europeans, South Asians, Others; http://exac.broadinstitute.org/), and gnomAD (African, American, Ashkenazi Jewish, East Asian, Finnish, Non-Finnish Europeans, South Asians, Others; http://gnomad.broadinstitute.org/).

We additionally analyzed genome sequencing data of 7,231 individuals with autism from the MSSNG study (https://www.mss.ng/#) for rare loss-of-function variants in genes of interest.

1. Disease variant databases

The Human Gene Mutation Database (HGMD; http://www.hgmd.cf.ac.uk/ac/index.php) (13) and ClinVar (https://www.ncbi.nlm.nih.gov/clinvar/) (14) were used as disease variant databases.

1. Sanger validation

All variants were confirmed by PCR and Sanger sequencing (primers and sequencing protocols are available upon request).

**Statistics**

We performed Fisher’s exact test for count data in R version 3.4.4 (number of subjects with absent pulmonary valve syndrome or right aortic arch, respectively, in the TOF subgroup of cohort 1).

**Table S1. Genome sequencing coverage statistics.**

| **Cohort 1 (230 samples)^a^** | **Mean coverage per genome** | **Proportion with coverage 10x or greater** | **Proportion with coverage 25x or greater** |
| --- | --- | --- | --- |
| **Average** | 40.93 | 0.98 | 0.93 |
| **Median** | 40.25 | 0.98 | 0.93 |
| **Range** | 27.68 - 172.41 | 0.93 - 0.98 | 0.67 - 0.98 |
| **Cohort 2 (27 samples)^b^** |  |  |  |
| **Average** | 37.07 | 0.98 | 0.91 |
| **Median** | 37.67 | 0.98 | 0.90 |
| **Range** | 32.40 - 39.78 | 0.97 - 0.98 | 0.85 - 0.95 |

^a^ One proband (TGA9, with transposition of the great arteries) of the n=231 in the adult cohort was an outlier with respect to coverage and thus omitted from the statistics presented in this table.

^b^ These samples comprise 10 affected probands, and one affected and 16 unaffected parents.

**Table S2. Summary descriptive statistics for 19 study participants with tetralogy of Fallot (TOF) and likely disruptive variants in vascular endothelial growth factor (VEGF) related signaling genes (cohort 1).^a^**

| **Demographics** | |
| --- | --- |
| Female : male | 10 : 9 |
| European ethnicity^b^ | 19/19 |
| Median age | 33 years |
| Completed secondary education | 17/19 |
| Studying, employed, or retired | 17/19 |
| **Cardiac phenotype** | |
| Median age at TOF repair | 4 years |
| Right aortic arch (RAA) | 10/19 |
| Absent pulmonary valve (APV) | 5/19 |
| PFO or ASD | 6/19 |
| **Family history** | |
| Congenital heart disease^c^ | 5/18 |
| Number of full siblings with CHD | 1/40 |
| Number of liveborn offspring with CHD | 2/17 |
| **Extracardiac phenotype** | |
| Short stature | 2/19 |
| Obesity | 3/19 |
| Abnormal head circumference | 0/19 |
| Intellectual disability | 0/19 |
| Learning difficulties | 5/19 |
| Depression and/or anxiety | 7/19 |
| Lymphedema | 0/19 |
| Hemangioma | 0/19 |
| Tumor history^d^ | 2/19 |
| Major congenital anomaly^e^ | 1/19 |

^a^ No significant differences in sex, age, learning difficulties. Significantly enriched for APV and RAA compared to the entire TOF cohort sequenced (n=175) (see text).

^b^ By design for the entire cohort (n=231; n=175 TOF) with sequencing data.

^c^ Known or strongly suspected congenital heart disease in any first to third degree relative: 5/18 with VEGF variant and family information available (versus 38/174 TOF probands, two-sided Fisher’s exact test: p=0.55). TOF293 had no family information available.

^d^ 79 year old with melanoma, 46 year old with benign brain tumor (Table 1): 2/19 with VEGF variant (versus 8/175 TOF probands, two-sided Fisher’s exact test: p=0.21).

^e^ Duplicated ureter (Table 1).

Abbreviations: ASD, atrial septal defect; CHD, congenital heart disease; PFO, patent foramen ovale; TOF, tetralogy of Fallot; VEGF, vascular endothelial growth factor.

**Table S3. Putative cardiac related variants of uncertain significance in 22 probands with TOF and likely disruptive variants in VEGF related signaling genes.**

| **Case^a^** | **Single nucleotide variants** | **Copy number variants (GRCh37/hg19)** | | | |
| --- | --- | --- | --- | --- | --- |
|  |  | **Size (kb)** | **Loss/Gain** | **Coordinates** | **Genes^b^ overlapped** |
| **Cohort 1 (n=19)** | | | | | |
| **Probands with *FLT4* variants (n=9)** | | | | | |
| **TOF293** | *SOS1* (NM_005633.3): c.670A>G, p.(Lys224Glu) |  |  |  |  |
| **TOF158** | *CHD4* (NM_001273.2): c.1475G>A, p.(Arg492His) |  |  |  |  |
| **TOF238** | *PLXND1* (NM_015103.2): c.2138C>T, p.(Ala713Val) | 152 | loss | chr17:47978986-48130937 | *DLX3* |
| **TOF284** |  |  |  |  |  |
| **TOF254** |  | 203 | gain | chr12:45942271-46145500 | *ARID2* exons 1-3 |
| **TOF68** |  | 24 | gain | chr19:34210751-34234569 | *CHST8* intron 3 |
| **TOF301** | *TLL1* (NM_012464.4): c.2996G>T, p.(Arg999Ile) |  |  |  |  |
| **TOF271** |  |  |  |  |  |
| **TOF236** |  |  |  |  |  |
| **Probands with *KDR* variants (n=4)** | | | | | |
| **TOF109** |  |  |  |  |  |
| **TOF155** | *RAF1* (NM_002880.3): c.1225A>G, p.(Met409Val) | 129 | loss | chr7:107653165-107782590 | *LAMB4* |
| **TOF326** |  |  |  |  |  |
| **TOF359** |  |  |  |  |  |
| **Probands with other VEGF variants (*VEGFA, FGD5, BCAR1, IQGAP1, FOXO1, PRDM1*, respectively)** | | | | | |
| **TOF241** |  |  |  |  |  |
| **TOF89** | *ECE1* (NM_001113349.1): c.868G>A, p.(Gly290Ser) |  |  |  |  |
| **TOF220** | *NOTCH1* (NM_017617.3): c.1869C>A, p.(Asn623Lys) | 337 | loss | chr7:11374706-11711740 | *THSD7A* exons 2-28 |
| **TOF48** |  | 26  221  254 | gain  gain  loss | chr10:88487754-88513897  chr8:53596811-53817643  chr18:63967846-64221878 | *LDB3* exon 13  *RB1CC1* exons 1-3  *CDH19* exons 5-12 |
| **TOF62** |  | 78 | gain | chr8:73708969-73787448 | *KCNB2* intron 2 |
| **TOF53** |  | 51 | loss | chr2:159252933-159304139 | *CCDC148* intron 1 |
| **Cohort 2 with *FLT4, KDR, IQGAP1* variants, respectively** | | | | | |
| **CGC-034** |  |  |  |  |  |
| **CGC-001^c^** | *NOTCH2* (NM_024408.3): c.4744G>A, p.(Asp1582Asn) |  |  |  |  |
| **CGC-076** | *CREBBP* (NM_004380.2):  c.5887G>A, p.(Glu1963Lys) |  |  |  |  |

^a^ See Table 1 for details.

^b^ Putative CHD relevant genes (1, 2).

^c^ CNV analysis from genome sequencing data only.

**
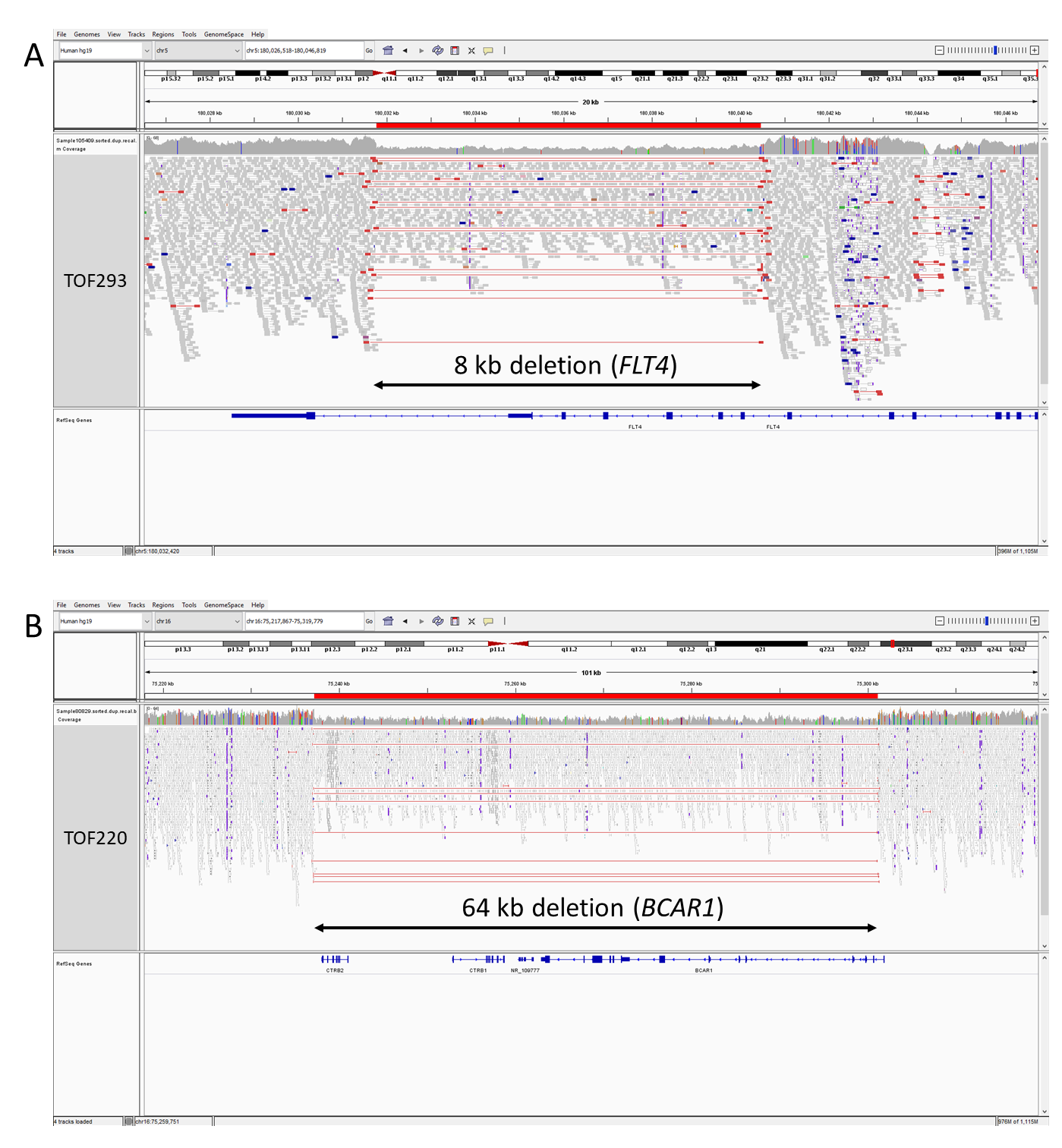
**

**Figure S1. Multi-exon deletions of *FLT4* (A) and *BCAR1* (B) identified by genome sequencing of individuals with TOF in the current study.** Representation of aligned sequence reads in the Integrative Genomics Viewer (IGV) (15, 16).

**Table S4. Copy number variants overlapping 15 VEGF pathway genes (*BCAR1*, *FGD5*, *FLT1*, *FLT4*, *FOXO1*, *IQGAP1*, *KDR*, *NRP1*, *NRP2*, *PGF*, *PRDM1*, *VEGFA*, *VEGFB*, *VEGFC, or VEGFD)* in individuals with cardiac defects from previous studies^a^.**

| **Gene(s)** | **Variant** | **Chromosomal position (GRCh37/hg19)** | **Study** | **Phenotype** | **Remarks** |
| --- | --- | --- | --- | --- | --- |
| ***FLT4***, *CNOT6*, *SCGB3A1* | 82 kb loss | chr5:179,985,934-180,067,675 | Xie et al. (17) | **TOF**; speech delay |  |
| ***FLT4*** | 29 kb loss | chr5:180,068,741-180,098,422 | Mak et al. (18) | **TOF**, absent left pulmonary artery, right pulmonary artery stenosis |  |
| ***FLT4*** | 31 kb gain | chr5:180,043,388-180,074,248 | Xie et al. (17) | **Aortic arch abnormality**; long philtrum, tented upper lip | Intragenic breakpoint |
| ***FOXO1***, *LINC00598* | 231 kb loss | chr13:41,039,186-41,270,080 | Xie et al. (17) | **TOF**; small palpebral fissures, broad nasal root, wide spaced nipples, jaundice – unconjugated; seizures |  |
| ***NRP1*** + 20 other genes | 7.2 Mb loss | chr10:28,688,677-35,865,291 | Goldmuntz et al. (19) | **VSD**; right hemifacial microsomia, microtia right ear, abnormal left inner ear, posterior embryotoxon, bilateral epicanthal folds, high narrow arched palate, tented lip; butterfly vertebrae |  |
| ***VEGFC*** + 23 other genes, including *HMGB2*, *SAP30*, *HAND2*, *HPGD*, *SPCS3*, *AGA* | 6.6 Mb loss | chr4: 173,538,77-6,551,32 | Soemedi et al. (20) | **TOF**; bilateral cryptorchidism; asthma | VEGFC pLI=0.12 |

**^a^** Reviewed studies: **(1, 2, 17-39).**

Abbreviations: TOF, tetralogy of Fallot; VSD, ventricular septal defect.

**Supplementary information on the development of the second heart field and vascular endothelial growth factor related genes.**

Four main progenitor populations give rise to cardiac structures: (i) the first heart field (FHF; left ventricle, most of the atria and a small part of the right ventricle), (ii) the second heart field (SHF; most of the right ventricle, outflow tract and a small part of the atria), arising from the splanchnic mesoderm (40), (iii) neural crest cells, and (iv) the proepicardial organ. The rate of proliferation of the SHF is very high (41).

Tetralogy of Fallot originates as a disease of the right ventricle and the outflow tract. The development of these structures occurs in four steps: (i) an influx of proliferating anterior SHF cells into the arterial pole of the heart tube, (ii) differentiation of these SHF cells into the right ventricle and outflow tract, (iii) entry of cardiac neural crest cells to septate the outflow tract, and (iv) rotation of the outflow tract to align the ventricles with the appropriate great artery. The outflow tract lies above the right ventricle and then rotates, the inferior component moves superiorly to form the subpulmonary myocardium and the original superior component moves from right to left to sit above the left ventricle and to the left of the interventricular septum. Defects in the anterior SHF can result in a hypoplastic right ventricular outflow tract, dysfunction of the cardiac neural crest can result in outflow tract malformations (including common arterial trunk) or SHF dysfunction. Rotational errors can lead to overriding aorta, double-outlet right ventricle and transposition of the great arteries.

VEGFR2 positive cells migrate from the primitive streak and contribute to the future pool of cardiomyocytes (42). Vascular endothelial growth factor signaling has multiple roles in the normal development of the SHF, from the proliferation and differentiation of progenitor cells (43), to the balance between growth of the endocardial cushions and the endocardial-to-mesenchymal transition (EMT) involved in semilunar valve formation (44). The EMT in the outflow tract involves VEGFR1, which is then downregulated, whereas VEGFR2 expression increases after EMT is complete and contributes to the differentiation of the atrioventricular cushions into atrioventricular valves, which also requires mir-126 (45).

**Vascular endothelial growth factors**

***VEGFA*:** *VEGFA* encodes vascular endothelial growth factor A (VEGFA). VEGFA is the best studied regulator of vascular development and permeability under physiological and pathological conditions (46). It stimulates endothelial cell migration and proliferation primarily through VEGFR2 (47). There are several splice variants of the protein, regulating the ability to bind and activate receptors. All isoforms bind VEGFR2, however only two isoforms (VEGF^165^ and VEGF^145^) bind VEGFR2/NRP1 complexes (44, 48). Several common variants in VEGFA and dysregulated VEGFA expression have been linked to cardiac development and TOF in humans (49-54). One of the main VEGFA downstream pathways is the NOTCH pathway, with several genes implicated in outflow tract development and syndromic/nonsyndromic congenital heart defects (e.g. *NOTCH1, NOTCH2, JAG1, DLL4*) (44).

***VEGFB*:** VEGFB is a selective VEGFR1 ligand, regulating fatty acid uptake in endothelial cells, but with otherwise restricted roles in angiogenesis *in vivo* (55, 56).

***VEGFC*:** VEGFC is a ligand predominantly of VEGFR3. It is a critical regulator of lymphendothelial cell functions, for example inducing migration and sprouting of lymphendothelial precursor cells (55).

***VEGFD*:** *VEGFD* encodes different isoforms of VEGFD, which can bind to VEGFR2 or VEGFR3, thereby exhibiting angiogenic or lymphangiogenic functions (57, 58).

***PGF*:** *PGF* (placental growth factor) encodes a selective VEGFR1 ligand, which appears not to be essential for physiological angiogenesis. However, *PGF* is involved in angiogenesis associated with ischemia, inflammation, wound healing and cancer (59).

**Vascular endothelial growth factor receptors**

***FLT1:*** *FLT1* encodes vascular endothelial growth factor receptor 1 (VEGFR1) a tyrosine kinase which can be activated by VEGFA, VEGFB and PGF. It is expressed in developing and adult vascular endothelial cells (60) and in a variety of non-endothelial cells, such as monocytes and macrophages (61). VEGFR1 can form heterodimers with VEGFR2 (62). The kinase activity of VEGFR1 is only weakly induced by ligand binding, and its role in endothelial cells is uncertain (55), it might have a role in endothelial cell differentiation and organization. The VEGFR1 protein may serve as a VEGF decoy, for example through sequestering of VEGFA (63). Alternative splicing results in the generation of a soluble form of the receptor (sVEGFR1/sFlt1) (64).

***KDR*:** *KDR* encodes vascular endothelial growth factor receptor tyrosine kinase 2 (VEGFR2), which is expressed most prominently in vascular endothelial cells during embryonic vasculogenesis and angiogenesis, but also in a variety of non-endothelial cells, for example in retinal progenitor cells, pancreatic duct cells and hematopoietic cells (65). VEGFR2 acts as a cell-surface receptor for VEGFA, VEGFC and VEGFD, and plays a pivotal role in angiogenesis, vascular development, vascular permeability, endothelial cell differentiation, proliferation, and migration (46, 47). *KDR*/VEGFR2 modulates *FLT1*/VEGFR1 and *FLT4*/VEGFR3 signaling by forming heterodimers (66, 67). Binding of vascular growth factors leads to the activation of several signaling cascades in endothelial cells, for example the phosphatidylinositol 3' (PI3K)-kinase/Akt pathway (68). Cytoskeletal remodeling and transcriptional regulation are downstream effectors of VEGFR2 signaling (69).

***FLT4*:** *FLT4* encodes vascular endothelial growth factor receptor tyrosine kinase 3 (VEGFR3). *FLT4* is expressed on developing venous endothelia and lymphatic vessels and acts as a cell-surface receptor, predominantly for VEGFC and VEGFD (70). *FLT4* plays an essential role in lymphangiogenesis and in embryonic cardiovascular development (71). Remarkably, early angiogenesis in mouse embryos was reported to be independent of VEGFC and VEGFD signaling (72), and neither required VEGFR3 kinase activity, indicating that VEGFR3 signaling is not directly required for angiogenesis, and its role in early cardiovascular development might be through modulating VEGFR2-mediated signals (73). VEGFA, VEGFC, and VEGFD were shown to induce the formation of VEGFR2/VEGFR3 heterodimers in developing blood vessels and lymphatic structures. Also, inhibition of such VEGFR2/VEGFR3 heterodimer formation decreased the extent of angiogenic sprouting (67, 74, 75).

***NRP1* and *NRP2*:** *NRP1* and *NRP2* encode VEGF coreceptors, transmembrane proteins lacking intrinsic catalytic functions (76). They were first identified as receptors for semaphorins, which are soluble molecules modulating the development of the nervous and vascular systems (77, 78). Certain VEGFA isoforms bind NRP1/VEGFR2 complexes (48), leading to enhanced endothelial cell migration and vascular permeability (79). The mechanism whereby NRP1 modulates VEGF biology is uncertain. A role of p130cas dependent pathway activation was reported (80). NRP2 associates with VEGFR3 and induces lymphatic sprouting in response to VEGFC or VEGFD signaling (81, 82).

**Vascular endothelial growth factor signaling (based on findings from this study)**

***BCAR1*:** *BCAR1* encodes p130Cas, an adaptor protein and kinase substrate. P130cas was shown to exhibit a key role in mediating VEGFR2/NRP1-dependent angiogenic signaling and endothelial cell migration (80, 83-85). P130cas is thought to function in the assembly of multiprotein signaling complexes required for the remodeling of the actin cytoskeleton during cell motility (83, 86). Both FGD5 and IQGAP1 were shown to be interaction partners of P130cas, and their association was increased by VEGFA signaling (83).

***FGD5*:** *FGD5* encodes a rho-family guanine nucleotide exchange factor, which is selectively expressed in endothelial cells (87). The protein forms a complex with VEGFR2 at the leading edge of the cell and among endosomes (69). Depletion of FGD5 in microvascular cells accelerated VEGFR2 degradation through proteasomal and lysosomal pathways, inhibited their migration towards a stable VEGFA gradient and reduced the number of angiogenic sprouts (69, 88). Knockdown of FGD5 also inhibited VEGFA induced activation of the Rho GTPase Cdc42 (87). FGD5 was shown to be involved in mTORC2-dependent cytoskeletal remodeling (69).

***FOXO1*:** *FOXO1* encodes a transcription factor, with a critical role in normal vascular development and the regulation of coordinated vascular sprouting. VEGF-regulated phosphorylation of VEGFR2 increased endothelial cell viability by suppressing the activity of FOXO1, with a central role of the PI3K-mTORC2-Akt axis (89). Endothelial-restricted deletion of FOXO1 in human cell cultures induced a profound increase in endothelial cell proliferation which interfered with coordinated sprouting, causing hyperplasia and vessel enlargement (90). In mice, Foxo1 is essential to the ability of endothelial cells to respond properly to VEGFA. Although vascular receptors were retained, endothelial cells appeared resistant to VEGFA (91). Foxo1(-/-) mouse endothelial cells could form only short sprouts, with disorganized cytoskeletal remodeling and absence of smooth muscle cell recruitment (92). In cancer cells, phosphorylated FOXO1 was also shown to bind IQGAP1, this interaction being regulated by the PI3K-Akt pathway (93).

***IQGAP1*:** *IQGAP1* encodes a GTPase-binding scaffold protein. *IQGAP1* is robustly expressed in endothelial cells, where it colocalized with active VEGFR2 at the leading edge of migrating endothelial cells (94). The association between VEGFR2, p130Cas and IQGAP1 in endothelial cells was increased by VEGFA, and was thought to play a key role in chemotactic signaling, potentially through Akt activation (83). IQGAP1 has been shown to regulate a number of essential cellular events. For example, IQGAP1 formed complexes with filamentous actin *in vivo*, and was required for Cdc42/Rac1-mediated actin polymerization *in vitro* (95). IQGAP1 knockdown inhibited angiogenesis, endothelial cell polarization, migration and proliferation (83, 94).

***PRDM1*:** *PRDM1* encodes a transcriptional repressor. In mice, *Prdm1* is expressed in the second heart field (96). *PRDM1* expression was shown to be upregulated by VEGF/VEGFR2 signalling in tumor vasculature and during wound healing. Under those conditions, a large proportion of VEGF-mediated transcriptional repressor activities were regulated by PRDM1/BF1 (97).

**Table S5. Mouse deficiency models of 15 VEGF pathway genes.**

| **Human gene** | **Mouse gene** | **Mouse model** |
| --- | --- | --- |
| ***FLT1*** | *Flt1* | Constitutive knock-out: Embryonic lethality (day 8.5-9), with normal endothelial cell differentiation but severely disorganized and dysfunctional vascular systems (98).  Flt-1 tyrosine kinase deficiency: Survival with normally developed vessels but suppressed macrophage migration (99).  Flt-1 transmembrane domain deficiency: 50% embryonic lethality with poorly developed blood vessels and small dorsal aortae. Heterozygous mice showed developmental defects similar to homozygous mice (100). |
| ***KDR*** | *Kdr* | Constitutive knock-out: Embryonic lethality (day 8.5-9.5), with early defects in the development of haematopoietic and endothelial cells (101). |
| ***FLT4*** | *Flt4* | Constitutive knock-out: Embryonic lethality (day 9.5), with abnormal vascular organization, fluid accumulation in the pericardial cavity, and cardiovascular failure (71).  Flt-4 tyrosine kinase or ligand binding deficiency: Normal VEGFR2 binding and normal blood angiogenesis, abnormal lymphangiogenesis (73). |
| ***NRP1*** | *Nrp1* | Constitutive knock-out: Embryonic lethality (day 10.5-12.5), with various defects of the heart, vasculature, and nervous system (102). |
| ***NRP2*** | *Nrp2* | Constitutive knock-out: Viable, but anomalies of the nervous system and lymphatic vessels (103, 104). |
| ***VEGFA*** | *Vegfa* | Constitutive knock-out: Embryonic lethality (day 10.5), with abnormally enlarged vascular structures and missing aorta (48).  Heterozygous inactivation: Embryonic lethality (day 11-12), with impaired cardiac and vessel development (48, 105, 106).  Half of newborn mice that lack the Nrp1-binding Vegfa^164^ isoform die of cardiac defects, many exhibiting TOF (107). |
| ***BCAR1*** | *Bcar1* | Constitutive knock-out: Embryonic lethality (day 11.5-12.5), with cardiovascular defects and dilated blood vessels (108). |
| ***FGD5*** | *Fgd5* | Constitutive knock-out: Embryonic lethality (day 11.5-12,) with perturbed angiogenesis and VEGF signaling (109, 110). |
| ***FOXO1*** | *Foxo1* | Constitutive knock-out: Embryonic lethality (day 11) with impaired vascular development in the yolk sacs and embryos, and underdevelopment of branchial arches. Endothelial cells differentiated from Foxo-/- embryonic stem cells showed abnormal response to exogenous VEGF (91). |
| ***IQGAP1*** | *Iqgap1* | Constitutive knock-out: Development appears normal, but with increase in late-onset gastric hyperplasia. Although IQGAP1-null mice have unaltered basal heart function, they exhibit unfavourable cardiac remodeling (thinning of left ventricular walls, chamber dilation, and a decrease in contractility) upon pressure overload (111, 112). |
| ***PRDM1*** | *Prdm1* | Conditional knock-out: Arterial pole defects, mis-alignment or reduction of the aorta and pulmonary trunk, and abnormalities in the arterial tree. Defects were preceded by a reduction in outflow tract size and loss of caudal pharyngeal arch arteries, and were even more pronounced on a Tbx1 heterozygote background (96). |
| ***VEGFB*** | *Vegfb* | Constitutive knock-out: Healthy and fertile, with decreased heart size and vascular dysfunction after coronary occlusion (113). |
| ***VEGFC*, *VEGFD* (*FIGF*)** | *Vegfc*, *Vegfd* (*Figf*) | Constitutive double-knock-out: Unaffected blood vessel formation, hypoplastic lymphatic vessels (72). |
| ***PGF*** | *Pgf* | Constitutive knock-out: Unaffected embryonic angiogenesis, but impaired angiogenesis during ischemia, inflammation, wound healing and cancer (59). |

**Supplementary literature**

1. Silversides CK, Lionel AC, Costain G, Merico D, Migita O, Liu B, et al. Rare copy number variations in adults with tetralogy of Fallot implicate novel risk gene pathways. PLoS Genet. 2012;8(8):e1002843.

2. Costain G, Lionel AC, Ogura L, Marshall CR, Scherer SW, Silversides CK, et al. Genome-wide rare copy number variations contribute to genetic risk for transposition of the great arteries. Int J Cardiol. 2016;204:115-21.

3. Li H, Durbin R. Fast and accurate short read alignment with Burrows-Wheeler transform. Bioinformatics. 2009;25(14):1754-60.

4. DePristo MA, Banks E, Poplin R, Garimella KV, Maguire JR, Hartl C, et al. A framework for variation discovery and genotyping using next-generation DNA sequencing data. Nat Genet. 2011;43(5):491-8.

5. Van der Auwera GA, Carneiro MO, Hartl C, Poplin R, Del Angel G, Levy-Moonshine A, et al. From FastQ data to high confidence variant calls: the Genome Analysis Toolkit best practices pipeline. Curr Protoc Bioinformatics. 2013;43:11 0 1-33.

6. Stavropoulos DJ, Merico D, Jobling R, Bowdin S, Monfared N, Thiruvahindrapuram B, et al. Whole Genome Sequencing Expands Diagnostic Utility and Improves Clinical Management in Pediatric Medicine. NPJ Genom Med. 2016;1.

7. Yuen RK, Thiruvahindrapuram B, Merico D, Walker S, Tammimies K, Hoang N, et al. Whole-genome sequencing of quartet families with autism spectrum disorder. Nat Med. 2015;21(2):185-91.

8. Wang K, Li M, Hakonarson H. ANNOVAR: functional annotation of genetic variants from high-throughput sequencing data. Nucleic Acids Res. 2010;38(16):e164.

9. Zhu M, Need AC, Han Y, Ge D, Maia JM, Zhu Q, et al. Using ERDS to infer copy-number variants in high-coverage genomes. Am J Hum Genet. 2012;91(3):408-21.

10. Abyzov A, Urban AE, Snyder M, Gerstein M. CNVnator: an approach to discover, genotype, and characterize typical and atypical CNVs from family and population genome sequencing. Genome Res. 2011;21(6):974-84.

11. Trost B, Walker S, Wang Z, Thiruvahindrapuram B, MacDonald JR, Sung WWL, et al. A Comprehensive Workflow for Read Depth-Based Identification of Copy-Number Variation from Whole-Genome Sequence Data. Am J Hum Genet. 2018;102(1):142-55.

12. Reuter MS, Walker S, Thiruvahindrapuram B, Whitney J, Cohn I, Sondheimer N, et al. The Personal Genome Project Canada: findings from whole genome sequences of the inaugural 56 participants. CMAJ. 2018;190(5):E126-E36.

13. Stenson PD, Ball EV, Mort M, Phillips AD, Shiel JA, Thomas NS, et al. Human Gene Mutation Database (HGMD): 2003 update. Hum Mutat. 2003;21(6):577-81.

14. Landrum MJ, Lee JM, Benson M, Brown G, Chao C, Chitipiralla S, et al. ClinVar: public archive of interpretations of clinically relevant variants. Nucleic Acids Res. 2016;44(D1):D862-8.

15. Robinson JT, Thorvaldsdottir H, Winckler W, Guttman M, Lander ES, Getz G, et al. Integrative genomics viewer. Nat Biotechnol. 2011;29(1):24-6.

16. Thorvaldsdottir H, Robinson JT, Mesirov JP. Integrative Genomics Viewer (IGV): high-performance genomics data visualization and exploration. Brief Bioinform. 2013;14(2):178-92.

17. Xie HM, Werner P, Stambolian D, Bailey-Wilson JE, Hakonarson H, White PS, et al. Rare copy number variants in patients with congenital conotruncal heart defects. Birth Defects Res. 2017;109(4):271-95.

18. Mak CCY, Chow PC, Liu APY, Chan KYK, Chu YWY, Mok GTK, et al. De novo large rare copy-number variations contribute to conotruncal heart disease in Chinese patients. NPJ Genom Med. 2016;1:16033.

19. Goldmuntz E, Paluru P, Glessner J, Hakonarson H, Biegel JA, White PS, et al. Microdeletions and microduplications in patients with congenital heart disease and multiple congenital anomalies. Congenit Heart Dis. 2011;6(6):592-602.

20. Soemedi R, Wilson IJ, Bentham J, Darlay R, Topf A, Zelenika D, et al. Contribution of global rare copy-number variants to the risk of sporadic congenital heart disease. Am J Hum Genet. 2012;91(3):489-501.

21. Thienpont B, Mertens L, de Ravel T, Eyskens B, Boshoff D, Maas N, et al. Submicroscopic chromosomal imbalances detected by array-CGH are a frequent cause of congenital heart defects in selected patients. Eur Heart J. 2007;28(22):2778-84.

22. Hitz MP, Lemieux-Perreault LP, Marshall C, Feroz-Zada Y, Davies R, Yang SW, et al. Rare copy number variants contribute to congenital left-sided heart disease. PLoS Genet. 2012;8(9):e1002903.

23. Lalani SR, Shaw C, Wang X, Patel A, Patterson LW, Kolodziejska K, et al. Rare DNA copy number variants in cardiovascular malformations with extracardiac abnormalities. Eur J Hum Genet. 2013;21(2):173-81.

24. Xie L, Chen JL, Zhang WZ, Wang SZ, Zhao TL, Huang C, et al. Rare de novo copy number variants in patients with congenital pulmonary atresia. PLoS One. 2014;9(5):e96471.

25. Zhao W, Niu G, Shen B, Zheng Y, Gong F, Wang X, et al. High-resolution analysis of copy number variants in adults with simple-to-moderate congenital heart disease. Am J Med Genet A. 2013;161A(12):3087-94.

26. White PS, Xie HM, Werner P, Glessner J, Latney B, Hakonarson H, et al. Analysis of chromosomal structural variation in patients with congenital left-sided cardiac lesions. Birth Defects Res A Clin Mol Teratol. 2014;100(12):951-64.

27. Greenway SC, Pereira AC, Lin JC, DePalma SR, Israel SJ, Mesquita SM, et al. De novo copy number variants identify new genes and loci in isolated sporadic tetralogy of Fallot. Nat Genet. 2009;41(8):931-5.

28. Priest JR, Girirajan S, Vu TH, Olson A, Eichler EE, Portman MA. Rare copy number variants in isolated sporadic and syndromic atrioventricular septal defects. Am J Med Genet A. 2012;158A(6):1279-84.

29. Warburton D, Ronemus M, Kline J, Jobanputra V, Williams I, Anyane-Yeboa K, et al. The contribution of de novo and rare inherited copy number changes to congenital heart disease in an unselected sample of children with conotruncal defects or hypoplastic left heart disease. Hum Genet. 2014;133(1):11-27.

30. Fakhro KA, Choi M, Ware SM, Belmont JW, Towbin JA, Lifton RP, et al. Rare copy number variations in congenital heart disease patients identify unique genes in left-right patterning. Proc Natl Acad Sci U S A. 2011;108(7):2915-20.

31. Tomita-Mitchell A, Mahnke DK, Struble CA, Tuffnell ME, Stamm KD, Hidestrand M, et al. Human gene copy number spectra analysis in congenital heart malformations. Physiol Genomics. 2012;44(9):518-41.

32. Breckpot J, Thienpont B, Arens Y, Tranchevent LC, Vermeesch JR, Moreau Y, et al. Challenges of interpreting copy number variation in syndromic and non-syndromic congenital heart defects. Cytogenet Genome Res. 2011;135(3-4):251-9.

33. Thorsson T, Russell WW, El-Kashlan N, Soemedi R, Levine J, Geisler SB, et al. Chromosomal Imbalances in Patients with Congenital Cardiac Defects: A Meta-analysis Reveals Novel Potential Critical Regions Involved in Heart Development. Congenit Heart Dis. 2015;10(3):193-208.

34. Kim DS, Kim JH, Burt AA, Crosslin DR, Burnham N, Kim CE, et al. Burden of potentially pathologic copy number variants is higher in children with isolated congenital heart disease and significantly impairs covariate-adjusted transplant-free survival. J Thorac Cardiovasc Surg. 2016;151(4):1147-51 e4.

35. Sanchez-Castro M, Eldjouzi H, Charpentier E, Busson PF, Hauet Q, Lindenbaum P, et al. Search for Rare Copy-Number Variants in Congenital Heart Defects Identifies Novel Candidate Genes and a Potential Role for FOXC1 in Patients With Coarctation of the Aorta. Circ Cardiovasc Genet. 2016;9(1):86-94.

36. An Y, Duan W, Huang G, Chen X, Li L, Nie C, et al. Genome-wide copy number variant analysis for congenital ventricular septal defects in Chinese Han population. BMC Med Genomics. 2016;9:2.

37. Glessner JT, Bick AG, Ito K, Homsy J, Rodriguez-Murillo L, Fromer M, et al. Increased frequency of de novo copy number variants in congenital heart disease by integrative analysis of single nucleotide polymorphism array and exome sequence data. Circ Res. 2014;115(10):884-96.

38. Carey AS, Liang L, Edwards J, Brandt T, Mei H, Sharp AJ, et al. Effect of copy number variants on outcomes for infants with single ventricle heart defects. Circ Cardiovasc Genet. 2013;6(5):444-51.

39. Bittel DC, Zhou XG, Kibiryeva N, Fiedler S, O'Brien JE, Jr., Marshall J, et al. Ultra high-resolution gene centric genomic structural analysis of a non-syndromic congenital heart defect, Tetralogy of Fallot. PLoS One. 2014;9(1):e87472.

40. Meilhac SM, Esner M, Kelly RG, Nicolas JF, Buckingham ME. The clonal origin of myocardial cells in different regions of the embryonic mouse heart. Dev Cell. 2004;6(5):685-98.

41. van den Berg G, Abu-Issa R, de Boer BA, Hutson MR, de Boer PA, Soufan AT, et al. A caudal proliferating growth center contributes to both poles of the forming heart tube. Circ Res. 2009;104(2):179-88.

42. Ema M, Takahashi S, Rossant J. Deletion of the selection cassette, but not cis-acting elements, in targeted Flk1-lacZ allele reveals Flk1 expression in multipotent mesodermal progenitors. Blood. 2006;107(1):111-7.

43. Moretti A, Caron L, Nakano A, Lam JT, Bernshausen A, Chen Y, et al. Multipotent embryonic isl1+ progenitor cells lead to cardiac, smooth muscle, and endothelial cell diversification. Cell. 2006;127(6):1151-65.

44. van den Akker NM, Caolo V, Molin DG. Cellular decisions in cardiac outflow tract and coronary development: an act by VEGF and NOTCH. Differentiation. 2012;84(1):62-78.

45. Stankunas K, Ma GK, Kuhnert FJ, Kuo CJ, Chang CP. VEGF signaling has distinct spatiotemporal roles during heart valve development. Dev Biol. 2010;347(2):325-36.

46. Gogat K, Le Gat L, Van Den Berghe L, Marchant D, Kobetz A, Gadin S, et al. VEGF and KDR gene expression during human embryonic and fetal eye development. Invest Ophthalmol Vis Sci. 2004;45(1):7-14.

47. Ferrara N, Gerber HP, LeCouter J. The biology of VEGF and its receptors. Nat Med. 2003;9(6):669-76.

48. Carmeliet P, Ferreira V, Breier G, Pollefeyt S, Kieckens L, Gertsenstein M, et al. Abnormal blood vessel development and lethality in embryos lacking a single VEGF allele. Nature. 1996;380(6573):435-9.

49. Sharma HS, Peters TH, Moorhouse MJ, van der Spek PJ, Bogers AJ. DNA microarray analysis for human congenital heart disease. Cell Biochem Biophys. 2006;44(1):1-9.

50. Peters TH, Sharma V, Yilmaz E, Mooi WJ, Bogers AJ, Sharma HS. DNA microarray and quantitative analysis reveal enhanced myocardial VEGF expression with stunted angiogenesis in human tetralogy of Fallot. Cell Biochem Biophys. 2013;67(2):305-16.

51. Li Q, Pan H, Guan L, Su D, Ma X. CITED2 mutation links congenital heart defects to dysregulation of the cardiac gene VEGF and PITX2C expression. Biochem Biophys Res Commun. 2012;423(4):895-9.

52. Lambrechts D, Devriendt K, Driscoll DA, Goldmuntz E, Gewillig M, Vlietinck R, et al. Low expression VEGF haplotype increases the risk for tetralogy of Fallot: a family based association study. J Med Genet. 2005;42(6):519-22.

53. Li X, Liu CL, Li XX, Li QC, Ma LM, Liu GL. VEGF Gene Polymorphisms are Associated with Risk of Tetralogy of Fallot. Med Sci Monit. 2015;21:3474-82.

54. Yan L, Ge Q, Xi C, Zhang X, Guo Y. Genetic variations of VEGF gene were associated with tetralogy of fallot risk in a Chinese Han population. Genet Test Mol Biomarkers. 2015;19(5):264-71.

55. Koch S, Tugues S, Li X, Gualandi L, Claesson-Welsh L. Signal transduction by vascular endothelial growth factor receptors. Biochem J. 2011;437(2):169-83.

56. Hagberg CE, Falkevall A, Wang X, Larsson E, Huusko J, Nilsson I, et al. Vascular endothelial growth factor B controls endothelial fatty acid uptake. Nature. 2010;464(7290):917-21.

57. Stacker SA, Stenvers K, Caesar C, Vitali A, Domagala T, Nice E, et al. Biosynthesis of vascular endothelial growth factor-D involves proteolytic processing which generates non-covalent homodimers. J Biol Chem. 1999;274(45):32127-36.

58. Leppanen VM, Jeltsch M, Anisimov A, Tvorogov D, Aho K, Kalkkinen N, et al. Structural determinants of vascular endothelial growth factor-D receptor binding and specificity. Blood. 2011;117(5):1507-15.

59. Carmeliet P, Moons L, Luttun A, Vincenti V, Compernolle V, De Mol M, et al. Synergism between vascular endothelial growth factor and placental growth factor contributes to angiogenesis and plasma extravasation in pathological conditions. Nat Med. 2001;7(5):575-83.

60. Peters KG, De Vries C, Williams LT. Vascular endothelial growth factor receptor expression during embryogenesis and tissue repair suggests a role in endothelial differentiation and blood vessel growth. Proc Natl Acad Sci U S A. 1993;90(19):8915-9.

61. Sawano A, Iwai S, Sakurai Y, Ito M, Shitara K, Nakahata T, et al. Flt-1, vascular endothelial growth factor receptor 1, is a novel cell surface marker for the lineage of monocyte-macrophages in humans. Blood. 2001;97(3):785-91.

62. Huang K, Andersson C, Roomans GM, Ito N, Claesson-Welsh L. Signaling properties of VEGF receptor-1 and -2 homo- and heterodimers. Int J Biochem Cell Biol. 2001;33(4):315-24.

63. Kappas NC, Zeng G, Chappell JC, Kearney JB, Hazarika S, Kallianos KG, et al. The VEGF receptor Flt-1 spatially modulates Flk-1 signaling and blood vessel branching. J Cell Biol. 2008;181(5):847-58.

64. Kendall RL, Thomas KA. Inhibition of vascular endothelial cell growth factor activity by an endogenously encoded soluble receptor. Proc Natl Acad Sci U S A. 1993;90(22):10705-9.

65. Oelrichs RB, Reid HH, Bernard O, Ziemiecki A, Wilks AF. NYK/FLK-1: a putative receptor protein tyrosine kinase isolated from E10 embryonic neuroepithelium is expressed in endothelial cells of the developing embryo. Oncogene. 1993;8(1):11-8.

66. Waltenberger J, Claesson-Welsh L, Siegbahn A, Shibuya M, Heldin CH. Different signal transduction properties of KDR and Flt1, two receptors for vascular endothelial growth factor. J Biol Chem. 1994;269(43):26988-95.

67. Nilsson I, Bahram F, Li X, Gualandi L, Koch S, Jarvius M, et al. VEGF receptor 2/-3 heterodimers detected in situ by proximity ligation on angiogenic sprouts. EMBO J. 2010;29(8):1377-88.

68. Gerber HP, McMurtrey A, Kowalski J, Yan M, Keyt BA, Dixit V, et al. Vascular endothelial growth factor regulates endothelial cell survival through the phosphatidylinositol 3'-kinase/Akt signal transduction pathway. Requirement for Flk-1/KDR activation. J Biol Chem. 1998;273(46):30336-43.

69. Farhan MA, Azad AK, Touret N, Murray AG. FGD5 regulates VEGF receptor-2 coupling to PI3 kinase and receptor recycling. Arterioscler Thromb Vasc Biol. 2017;37(12):2301-10.

70. Kaipainen A, Korhonen J, Mustonen T, van Hinsbergh VW, Fang GH, Dumont D, et al. Expression of the fms-like tyrosine kinase 4 gene becomes restricted to lymphatic endothelium during development. Proc Natl Acad Sci U S A. 1995;92(8):3566-70.

71. Dumont DJ, Jussila L, Taipale J, Lymboussaki A, Mustonen T, Pajusola K, et al. Cardiovascular failure in mouse embryos deficient in VEGF receptor-3. Science. 1998;282(5390):946-9.

72. Haiko P, Makinen T, Keskitalo S, Taipale J, Karkkainen MJ, Baldwin ME, et al. Deletion of vascular endothelial growth factor C (VEGF-C) and VEGF-D is not equivalent to VEGF receptor 3 deletion in mouse embryos. Mol Cell Biol. 2008;28(15):4843-50.

73. Zhang L, Zhou F, Han W, Shen B, Luo J, Shibuya M, et al. VEGFR-3 ligand-binding and kinase activity are required for lymphangiogenesis but not for angiogenesis. Cell Res. 2010;20(12):1319-31.

74. Alam A, Herault JP, Barron P, Favier B, Fons P, Delesque-Touchard N, et al. Heterodimerization with vascular endothelial growth factor receptor-2 (VEGFR-2) is necessary for VEGFR-3 activity. Biochem Biophys Res Commun. 2004;324(2):909-15.

75. Tvorogov D, Anisimov A, Zheng W, Leppanen VM, Tammela T, Laurinavicius S, et al. Effective suppression of vascular network formation by combination of antibodies blocking VEGFR ligand binding and receptor dimerization. Cancer Cell. 2010;18(6):630-40.

76. Fujisawa H, Kitsukawa T, Kawakami A, Takagi S, Shimizu M, Hirata T. Roles of a neuronal cell-surface molecule, neuropilin, in nerve fiber fasciculation and guidance. Cell Tissue Res. 1997;290(2):465-70.

77. He Z, Tessier-Lavigne M. Neuropilin is a receptor for the axonal chemorepellent Semaphorin III. Cell. 1997;90(4):739-51.

78. Kolodkin AL, Levengood DV, Rowe EG, Tai YT, Giger RJ, Ginty DD. Neuropilin is a semaphorin III receptor. Cell. 1997;90(4):753-62.

79. Wang L, Zeng H, Wang P, Soker S, Mukhopadhyay D. Neuropilin-1-mediated vascular permeability factor/vascular endothelial growth factor-dependent endothelial cell migration. J Biol Chem. 2003;278(49):48848-60.

80. Evans IM, Yamaji M, Britton G, Pellet-Many C, Lockie C, Zachary IC, et al. Neuropilin-1 signaling through p130Cas tyrosine phosphorylation is essential for growth factor-dependent migration of glioma and endothelial cells. Mol Cell Biol. 2011;31(6):1174-85.

81. Karpanen T, Heckman CA, Keskitalo S, Jeltsch M, Ollila H, Neufeld G, et al. Functional interaction of VEGF-C and VEGF-D with neuropilin receptors. FASEB J. 2006;20(9):1462-72.

82. Xu Y, Yuan L, Mak J, Pardanaud L, Caunt M, Kasman I, et al. Neuropilin-2 mediates VEGF-C-induced lymphatic sprouting together with VEGFR3. J Cell Biol. 2010;188(1):115-30.

83. Evans IM, Kennedy SA, Paliashvili K, Santra T, Yamaji M, Lovering RC, et al. Vascular endothelial growth factor (VEGF) promotes assembly of the p130Cas interactome to drive endothelial chemotactic signaling and angiogenesis. Mol Cell Proteomics. 2017;16(2):168-80.

84. Pellet-Many C, Frankel P, Evans IM, Herzog B, Junemann-Ramirez M, Zachary IC. Neuropilin-1 mediates PDGF stimulation of vascular smooth muscle cell migration and signalling via p130Cas. Biochem J. 2011;435(3):609-18.

85. Endo A, Fukuhara S, Masuda M, Ohmori T, Mochizuki N. Selective inhibition of vascular endothelial growth factor receptor-2 (VEGFR-2) identifies a central role for VEGFR-2 in human aortic endothelial cell responses to VEGF. J Recept Signal Transduct Res. 2003;23(2-3):239-54.

86. Barrett A, Pellet-Many C, Zachary IC, Evans IM, Frankel P. p130Cas: a key signalling node in health and disease. Cell Signal. 2013;25(4):766-77.

87. Kurogane Y, Miyata M, Kubo Y, Nagamatsu Y, Kundu RK, Uemura A, et al. FGD5 mediates proangiogenic action of vascular endothelial growth factor in human vascular endothelial cells. Arterioscler Thromb Vasc Biol. 2012;32(4):988-96.

88. Heldin J, O'Callaghan P, Hernandez Vera R, Fuchs PF, Gerwins P, Kreuger J. FGD5 sustains vascular endothelial growth factor A (VEGFA) signaling through inhibition of proteasome-mediated VEGF receptor 2 degradation. Cell Signal. 2017;40:125-32.

89. Zhuang G, Yu K, Jiang Z, Chung A, Yao J, Ha C, et al. Phosphoproteomic analysis implicates the mTORC2-FoxO1 axis in VEGF signaling and feedback activation of receptor tyrosine kinases. Sci Signal. 2013;6(271):ra25.

90. Wilhelm K, Happel K, Eelen G, Schoors S, Oellerich MF, Lim R, et al. FOXO1 couples metabolic activity and growth state in the vascular endothelium. Nature. 2016;529(7585):216-20.

91. Furuyama T, Kitayama K, Shimoda Y, Ogawa M, Sone K, Yoshida-Araki K, et al. Abnormal angiogenesis in Foxo1 (Fkhr)-deficient mice. J Biol Chem. 2004;279(33):34741-9.

92. Park SH, Sakamoto H, Tsuji-Tamura K, Furuyama T, Ogawa M. Foxo1 is essential for in vitro vascular formation from embryonic stem cells. Biochem Biophys Res Commun. 2009;390(3):861-6.

93. Pan CW, Jin X, Zhao Y, Pan Y, Yang J, Karnes RJ, et al. AKT-phosphorylated FOXO1 suppresses ERK activation and chemoresistance by disrupting IQGAP1-MAPK interaction. EMBO J. 2017;36(8):995-1010.

94. Yamaoka-Tojo M, Ushio-Fukai M, Hilenski L, Dikalov SI, Chen YE, Tojo T, et al. IQGAP1, a novel vascular endothelial growth factor receptor binding protein, is involved in reactive oxygen species--dependent endothelial migration and proliferation. Circ Res. 2004;95(3):276-83.

95. Malarkannan S, Awasthi A, Rajasekaran K, Kumar P, Schuldt KM, Bartoszek A, et al. IQGAP1: a regulator of intracellular spacetime relativity. J Immunol. 2012;188(5):2057-63.

96. Vincent SD, Mayeuf-Louchart A, Watanabe Y, Brzezinski JAt, Miyagawa-Tomita S, Kelly RG, et al. Prdm1 functions in the mesoderm of the second heart field, where it interacts genetically with Tbx1, during outflow tract morphogenesis in the mouse embryo. Hum Mol Genet. 2014;23(19):5087-101.

97. Arulanandam R, Batenchuk C, Angarita FA, Ottolino-Perry K, Cousineau S, Mottashed A, et al. VEGF-mediated induction of PRD1-BF1/Blimp1 expression sensitizes tumor vasculature to oncolytic virus infection. Cancer Cell. 2015;28(2):210-24.

98. Fong GH, Rossant J, Gertsenstein M, Breitman ML. Role of the Flt-1 receptor tyrosine kinase in regulating the assembly of vascular endothelium. Nature. 1995;376(6535):66-70.

99. Hiratsuka S, Minowa O, Kuno J, Noda T, Shibuya M. Flt-1 lacking the tyrosine kinase domain is sufficient for normal development and angiogenesis in mice. Proc Natl Acad Sci U S A. 1998;95(16):9349-54.

100. Hiratsuka S, Nakao K, Nakamura K, Katsuki M, Maru Y, Shibuya M. Membrane fixation of vascular endothelial growth factor receptor 1 ligand-binding domain is important for vasculogenesis and angiogenesis in mice. Mol Cell Biol. 2005;25(1):346-54.

101. Shalaby F, Rossant J, Yamaguchi TP, Gertsenstein M, Wu XF, Breitman ML, et al. Failure of blood-island formation and vasculogenesis in Flk-1-deficient mice. Nature. 1995;376(6535):62-6.

102. Kawasaki T, Kitsukawa T, Bekku Y, Matsuda Y, Sanbo M, Yagi T, et al. A requirement for neuropilin-1 in embryonic vessel formation. Development. 1999;126(21):4895-902.

103. Giger RJ, Cloutier JF, Sahay A, Prinjha RK, Levengood DV, Moore SE, et al. Neuropilin-2 is required in vivo for selective axon guidance responses to secreted semaphorins. Neuron. 2000;25(1):29-41.

104. Yuan L, Moyon D, Pardanaud L, Breant C, Karkkainen MJ, Alitalo K, et al. Abnormal lymphatic vessel development in neuropilin 2 mutant mice. Development. 2002;129(20):4797-806.

105. Ferrara N, Carver-Moore K, Chen H, Dowd M, Lu L, O'Shea KS, et al. Heterozygous embryonic lethality induced by targeted inactivation of the VEGF gene. Nature. 1996;380(6573):439-42.

106. van den Akker NM, Molin DG, Peters PP, Maas S, Wisse LJ, van Brempt R, et al. Tetralogy of fallot and alterations in vascular endothelial growth factor-A signaling and notch signaling in mouse embryos solely expressing the VEGF120 isoform. Circ Res. 2007;100(6):842-9.

107. Stalmans I, Lambrechts D, De Smet F, Jansen S, Wang J, Maity S, et al. VEGF: a modifier of the del22q11 (DiGeorge) syndrome? Nat Med. 2003;9(2):173-82.

108. Honda H, Oda H, Nakamoto T, Honda Z, Sakai R, Suzuki T, et al. Cardiovascular anomaly, impaired actin bundling and resistance to Src-induced transformation in mice lacking p130Cas. Nat Genet. 1998;19(4):361-5.

109. Gazit R, Mandal PK, Ebina W, Ben-Zvi A, Nombela-Arrieta C, Silberstein LE, et al. Fgd5 identifies hematopoietic stem cells in the murine bone marrow. J Exp Med. 2014;211(7):1315-31.

110. Cheng C, Haasdijk R, Tempel D, van de Kamp EH, Herpers R, Bos F, et al. Endothelial cell-specific FGD5 involvement in vascular pruning defines neovessel fate in mice. Circulation. 2012;125(25):3142-58.

111. Li S, Wang Q, Chakladar A, Bronson RT, Bernards A. Gastric hyperplasia in mice lacking the putative Cdc42 effector IQGAP1. Mol Cell Biol. 2000;20(2):697-701.

112. Sbroggio M, Carnevale D, Bertero A, Cifelli G, De Blasio E, Mascio G, et al. IQGAP1 regulates ERK1/2 and AKT signalling in the heart and sustains functional remodelling upon pressure overload. Cardiovasc Res. 2011;91(3):456-64.

113. Bellomo D, Headrick JP, Silins GU, Paterson CA, Thomas PS, Gartside M, et al. Mice lacking the vascular endothelial growth factor-B gene (Vegfb) have smaller hearts, dysfunctional coronary vasculature, and impaired recovery from cardiac ischemia. Circ Res. 2000;86(2):E29-35.
